# Transient Expression and Immunogenicity Assessment of the *Dermatophagoides pteronyssinus* Der p 2 Allergen Produced in *Nicotiana benthamiana*

**DOI:** 10.3390/vaccines14030256

**Published:** 2026-03-11

**Authors:** Kotchaporn Jirananon, Kanokporn Thiganta, Kaewta Rattanapisit, Balamurugan Shanmugaraj, Waranyoo Phoolcharoen

**Affiliations:** 1 Baiya Phytopharm Co., Ltd., Bangkok 10330, Thailand; kotchaporn.j@baiyaphytopharm.com (K.J.); kanokporn.t@baiyaphytopharm.com (K.T.); kaewta.r@baiyaphytopharm.com (K.R.); 2 M.Sc. Program in Research for Enterprise, Faculty of Pharmaceutical Sciences, Chulalongkorn University, Bangkok 10330, Thailand; 3 Centre for Natural Products and Functional Foods, Karpagam Academy of Higher Education, Coimbatore 641021, Tamil Nadu, India; 4 Department of Biotechnology, Karpagam Academy of Higher Education, Coimbatore 641021, Tamil Nadu, India; 5 Center of Excellence in Plant-Produced Pharmaceuticals, Chulalongkorn University, Bangkok 10330, Thailand; 6 Department of Pharmacognosy and Pharmaceutical Botany, Faculty of Pharmaceutical Sciences, Chulalongkorn University, Bangkok 10330, Thailand

**Keywords:** recombinant protein, vaccine, house dust mite allergy, *Dermatophagoides pteronyssinus*, transient expression, *Nicotiana benthamiana*

## Abstract

Background: House dust mites (HDM) are one of the significant indoor allergen sources which cause IgE-mediated responses in most of the allergic individuals. HDMs are found in human habitats worldwide and Der p 2 is one of the major clinically relevant HDM allergens involved in triggering allergic diseases. The recombinant production of Der p 2 in plant systems provides a cost-effective and viable platform for developing diagnostic kits and allergen-specific immunotherapy. Methods: The *D. pteronyssinus* Der p 2 allergen was transiently expressed in *Nicotiana benthamiana* and its immunogenicity was evaluated in mice. The Der p 2 coding sequence was cloned into a geminiviral plant expression vector and introduced into *N. benthamiana* leaves via *Agrobacterium tumefaciens*-mediated infiltration. Recombinant Der p 2 proteins were purified from the crude extracts and confirmed by sodium dodecyl sulfate–polyacrylamide gel electrophoresis and Western blot. The immunogenicity of the plant-produced Der p 2 proteins was further evaluated by immunizing mice following a prime–boost immunization regimen, and Der p 2-specific antibody responses were assessed by ELISA. Results: Recombinant Der p 2 was successfully expressed and purified from *N. benthamiana*, and immunized mice developed high levels of Der p 2-specific IgG antibodies, with antibody titers increased after booster immunization. Conclusions: The results demonstrate that the transient expression of Der p 2 in plants is a feasible and effective strategy for producing immunologically active recombinant allergen proteins for diagnostic and potential clinical applications.

## 1. Introduction

House dust mites (HDM) are commonly found in indoor environments and represent the common sources of indoor aeroallergens that cause allergic disorders including perennial allergic rhinitis, allergic asthma and atopic dermatitis [1,2]. Among the various HDM species, *Dermatophagoides pteronyssinus* contributes significantly to aeroallergen exposure in human environments, whose protein components are believed to be the key contributors to allergies. The *D. pteronyssinus* allergen, Der p 2, is one of the most clinically relevant proteins in HDM allergy, with up to 90% of mite-allergic individuals exhibiting IgE sensitization to this allergen. Hence, Der p 2 is considered as a key target for both diagnostic applications and allergen-specific immunotherapy [3,4,5,6].

The allergen-specific immunotherapies primarily based on the repeated administration of crude allergen extracts with the aim to induce protective immune response [7]. The major problem of extract-based allergen-specific immunotherapy is these preparations are heterogeneous and may contain several other components along with major allergens which sometimes cause severe side effects [8]. Further, this variability complicates the batch-to-batch consistency, dosing, standardization, safety and consistency of treatment outcomes. These bottlenecks can be overcome by utilizing recombinant technologies and molecular approaches [9,10]. The advancement of recombinant DNA technology has enabled the production of highly purified recombinant allergens that can closely replicate the immunologically relevant epitope profiles of their natural counterparts. The recombinant allergen-based vaccines have been evaluated in clinical studies and have demonstrated encouraging clinical efficacy, along with improved safety profiles in allergic patients [11,12,13,14,15]. Thus recombinant allergen-based immunotherapies have emerged as promising alternatives to conventional therapy due to their defined molecular composition, improved reproducibility, and potential for tailored immune modulation [16].

Within this context, the recombinant Der p 2 has been extensively studied due to its immunodominance and clinical relevance in HDM-sensitized individuals [3,17]. However, the efficient utilization of recombinant Der p 2 in both diagnostic and therapeutic settings depends critically on the availability of efficient and reliable protein production systems capable of producing high-quality, clinical-grade allergens. Hence, there remains a need for efficient, rapid protein production systems that retain native conformational epitopes and immunological activity of Der p 2.

Recently, plant-based expression systems have emerged as an alternative platform for the production of vaccine antigens and other biopharmaceuticals. Plants offer several advantages which were highlighted in hundreds of earlier studies, including cost-effectiveness, scalability, low risk of human pathogen contamination, and the ability to perform complex post-translational modifications [18,19,20,21,22,23,24]. The transient expression of recombinant proteins in tobacco plants has gained considerable attention as a rapid and flexible platform in the recent years. Agroinfiltration-based transient expression when combined with geminiviral vectors enables high accumulation of recombinant proteins within a matter of days. This approach has been successfully applied for the production of vaccine antigens, monoclonal antibodies, antimicrobial peptides, diagnostic reagents, growth factors and other high-value proteins making it well-suited for the production of clinical-grade pharmaceutically relevant proteins [25,26,27,28,29].

The recombinant allergens have been produced in the plant system earlier. Burtin et al. demonstrated the feasibility of producing both native and genetically modified forms of the major HDM allergen Der p 1 from *D. pteronyssinus* in tobacco plants. The plant-derived native rDer p 1 displayed expected molecular weight and maintained IgE-binding capacity when tested with sera from allergic patients, confirming preservation of relevant conformational epitopes [30]. Similarly, Lee et al. demonstrated that the transgenic tobacco plants expressing Der p 2 can elicitoral tolerance in mice, resulting in reduced allergen-specific IgE responses, suppression of cytokines, and attenuation of airway inflammation [31]. However, the development of stable transgenic lines is time-consuming, labor-intensive and may result in low protein yields. In another study, a recombinant chimeric allergen (R8) derived from the major group 1 allergens of *Dermatophagoides farinae* and *Dermatophagoides pteronyssinus* was produced in *N. benthamiana* using the tobacco mosaic virus RNA-based overexpression vector. The plant-produced R8 retained IgE-binding capacity [32]. Additionally, the *Dermatophagoides farina* Der f1 and Der f2 proteins were expressed in *E. coli* and *N. benthamiana* plants further confirming the feasibility of plant-based production of recombinant mite allergens [33]. In the present study, we report the transient expression of Der p 2 fused to the human Immunoglobulin G1 (IgG1) Fc domain in *N. benthamiana* using a geminiviral expression system. The integrity and immunogenic potential of the plant-produced recombinant proteins were also evaluated. This work laid a foundation for the rapid production of plant-based recombinant Der p 2 as a well-defined component for future HDM allergen-specific immunotherapy and potential preventive immunization strategies.

## 2. Materials and Methods

### 2.1. Construction of Plant Expression Vector

**Figure 1:**
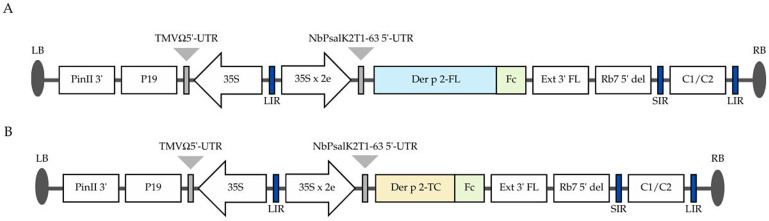
Schematic representation of the geminiviral plant expression vector (pBYR2e) used for the expression of Der p 2 fusion proteins in *N. benthamiana*. (**A**) T-DNA region of the pBYR2e vector carrying the full-length Der p 2 fused to the human IgG1 Fc fragment (Der p 2-FL-Fc). (**B**) T-DNA region of the same pBYR2e vector carrying a truncated Der p 2 variant lacking the N-terminal region of the mature protein, fused to the human IgG1 Fc fragment (Der p 2-TC-Fc).

The *D. pteronyssinus* Der p 2 gene sequence used in this study corresponds to the standardized IUIS allergen isoform Der p 2.0101 (UniProtKB Accession Number P49278, GenBank nucleotide: AF276239). The Der p 2 coding sequence was genetically fused with Fc region of human IgG1 (UniProtKB Accession Number: P01857) at the *C*-terminus. In this study, two plant expression constructs were generated, *viz.*, full-length Der p 2 fused to the Fc fragment (Der p 2-FL-Fc) and a truncated variant (Der p 2-TC-Fc) which lacks the *N*-terminal region of the mature protein (residue 1–51 of P49278), fused to the Fc fragment. The truncation boundary was defined based on previously reported epitope mapping studies [34,35]. Both the gene constructs were cloned into the plant expression vector pBYR2eK2Md (pBYR2e) [36]. The human Fc region was digested with *Bam*HI/*Sac*I (New England Biolabs, Ipswich, MA, USA) and ligated into the geminiviral expression vector containing the respective Der p 2 constructs using *Xba*I/*Sac*I (New England Biolabs, Ipswich, MA, USA) for plant expression. Figure 1 illustrates the schematic representation of the vector constructs used in the present work.

### 2.2. Protein Expression and Purification

The plant expression constructs pBYR2e harboring either Der p 2-FL-Fc and Der p 2-TC-Fc were electroporated into *Agrobacterium tumefaciens* strain GV3101 using the MicroPulser (Bio-Rad, Hercules, CA, USA). The putative transformants were confirmed by colony PCR using the gene-specific forward primers (nSP-Der2-Fu-F: 5′-GCTACCGGCGTTCACTCTGATTGGACCTGGATCCTG and nSP-Der2-TC-F: 5′-GCTACCGGCGTTCACTCTGACTGGACCTGGATCCTG) and reverse primer (78R: GCTTTGCATTCTTGACATC). *A. tumefaciens* carrying the expression constructs was infiltrated into the *N. benthamiana* leaves at a final OD_600_ of 0.2. The infiltrated leaves were collected at 2, 4, 6 and 8 days post-infiltration (dpi). For expression analysis, leaf tissues from independently infiltrated plants were pooled at each time point, weighed and homogenized in phosphate-buffered saline (PBS) at 1:2 (*w*/*v*) ratio. The crude extracts were clarified by centrifugation, and the total soluble protein (TSP) was determined using the Bradford assay (Bio-Rad, USA), and the protein yield was quantified by ELISA (Greiner Bio-One, Frickenhausen, Germany). The plant-derived protein was purified, concentrated and examined by sodium dodecyl sulfate–polyacrylamide gel electrophoresis (SDS–PAGE) and Western blot by following established protocols [37].

For Western blot analysis, equal amount of TSP (8 µg per lane) was separated in 8–12% polyacrylamide gel under reducing conditions and transferred to nitrocellulose membrane (Bio-Rad, USA). The membrane was blocked with 5% skim milk in PBS-T (0.05% Tween-20) and incubated with goat anti-human IgG Fc conjugated with HRP (SouthernBiotech, Birmingham, AL, USA), diluted at the ratio of 1:5000. The protein bands were visualized using enhanced chemiluminescence (ECL) substrate (ThermoFisher Scientific, Waltham, MA, USA). The relative protein accumulation was assessed by densitometry using ImageQuant TL software v.10.0 (Cytiva, Marlborough, MA, USA), and the relative expression levels were normalized to the 2 dpi time point [37,38].

For biochemical characterization, recombinant Fc-fusion proteins were purified from clarified extracts using protein A affinity chromatography (MabSelectPrismA, Marlborough, MA, USA, Cytiva). The purified proteins were analyzed by SDS–PAGE and Western blot as described above to confirm the expected molecular weight.

### 2.3. Mice Immunization

All animal experiments were conducted in accordance with institutional guidelines and were approved by the relevant institutional animal ethics committee (Approval number: 2491032). Female BALB/c mice (4–6 weeks old) were randomly divided into three groups (*n* = 5 per group). The control group received phosphate-buffered saline (PBS). On days 0 and 21, mice were intramuscularly (IM) administered with 10 µg of either plant-produced Der p 2-FL-Fc or Der p 2-TC-Fc in a total injection volume of 50 µL, including10 µL of alum adjuvant, and sera were collected on days 0 (pre-immune), 14, and 35. The Der p 2-specific antibody responses in the collected sera were evaluated by ELISA.

### 2.4. Evaluation of Immunological Responses in Mice

Der p 2-specific IgG, IgG1, and IgG2a levels were quantified by indirect ELISA. Briefly, 96-well plates (Greiner Bio-One, Frickenhausen, Germany) were coated with 2 µg/mL of recombinant Der p 2.0103 without an Fc tag (InBio, Charlottesville, VA, USA) in PBS (pH 7.4) and incubated overnight at 4 °C. The plates were washed three times with PBS-T (0.05% *v*/*v* Tween-20) and blocked with 5% skim milk in PBS for 2 h at 37 °C. Serial two-fold dilutions of mouse sera, starting from 1:100, were added and incubated for 2 h at 37 °C. Subsequently, antigen-specific antibodies were detected using HRP-conjugated goat anti-mouse IgG (Jackson ImmunoResearch, West Grove, PA, USA), IgG1 (Abcam, Cambridge, UK), or IgG2a (Abcam, Cambridge, UK), at a dilution of 1:2000 in PBS, and incubated for 1 h at 37 °C. The reaction was developed using 3,3′,5,5′-tetramethylbenzidine (TMB) substrate solution (Promega, Madison, WI, USA) and stopped with 1M H_2_SO_4_ (RCI labscan, Bangkok, Thailand). The optical density was measured at 450 nm using a microplate reader (Molecular Devices, San Jose, CA, USA). The endpoint titers were defined as the reciprocal of the highest serum dilution yielding an OD_450_ value greater than the cutoff value (mean OD_450_ of pre-immune sera +2SD). All samples were assayed in duplicates.

### 2.5. Statistical Analysis

Data are presented as mean ± SD. The statistical analyses were performed using IBM SPSS Statistics (Version 30). The differences in antibody responses were analyzed using two-way repeated-measures analysis of variance (ANOVA) with time (day 14 and day 35) as the within-subject factor and construct (FL-Fc vs. TC-Fc) as the between-subject factor. Prior to analysis, antibody titers were log10-transformed to meet the assumptions of normality. When significant main or interaction effects were observed, Bonferroni-adjusted pairwise comparisons were conducted. A *p*-value < 0.05 was considered statistically significant.

## 3. Results

### 3.1. HDM Der P 2-Fc Expression in N. Benthamiana

**Figure 2:**
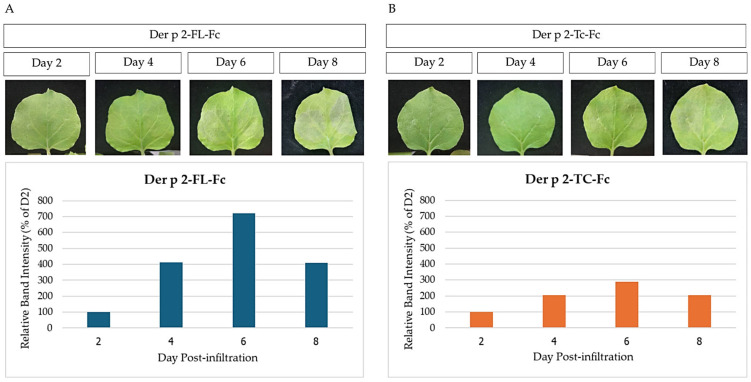
Optimization of Der p 2 expression in *N. benthamiana*. (**A**) Der p 2-FL-Fc; (**B**) Der p 2-Tc-Fc. Development of necrosis in *Agrobacterium*-infiltrated leaves at 2, 4, 6, and 8 dpi. Localized necrotic symptoms were observed at the infiltration sites. Representative Western blot analysis showing recombinant protein accumulation in crude leaf extracts at 2, 4, 6, and 8 dpi. Densitometric values were obtained from pooled samples and normalized to the 2 dpi time point.

The HDM Der p 2-FL-Fc and Der p 2-TC-Fc fusion constructs were cloned into the geminiviral plant expression vector pBYR2e (Figure 1) and introduced into *Agrobacterium tumefaciens*. The recombinant *Agrobacterium* harboring either one of these constructs was agroinfiltrated into *N. benthamiana* leaves. Both Der p 2-FL-Fc and Der p 2-TC-Fc constructs exhibited necrosis at the infiltration sites compared to control plants (Figure 2A,B upper panels). The preliminary expression kinetics was assessed by collecting the infiltrated leaves on 2, 4, 6 and 8 dpi. Recombinant protein accumulation was assessed by Western blot analysis followed by densitometric quantification using ImageQuant TL software (Cytiva). Densitometric values were obtained from pooled samples at each time point and used for relative comparison (detailed densitometric analysis, including ROIs and intensity graphs, is available in Supplementary Figure S1). The time-course analysis revealed that the expression of both Der p 2-FL-Fc and Der p 2-TC-Fc increased after infiltration, reaching maximal levels at 6 dpi (Figure 2A,B, lower panels; original uncropped blots are provided in Supplementary Figure S1). At 6 dpi, purified recombinant protein yield reached up to 62.4 µg/g fresh leaf weight for Der p2-FL-Fc and 15.5 µg/g fresh leaf weight for Der p2-TC-Fc as determined by ELISA. A decline in protein accumulation was observed at 8 dpi, which might be due to protein degradation associated with tissue necrosis [39,40].

### 3.2. Purification and Characterization of Plant-Produced Protein

**Figure 3:**
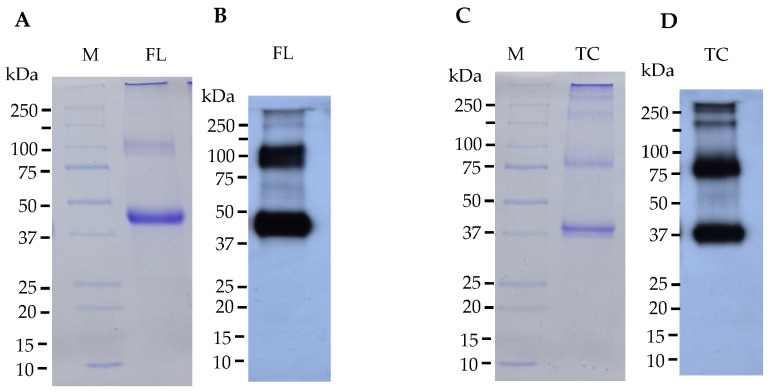
SDS–PAGE and Western blot analysis of plant-produced Der p 2–Fc fusion proteins expressed in *N. benthamiana* under reducing conditions. (**A**) SDS–PAGE analysis of purified Der p 2–FL–Fc stained with Coomassie Blue (Abcam, UK). (**B**) Western blot detection of Der p 2–FL–Fc using anti-human IgG Fc antibody. (**C**) SDS–PAGE analysis of purified Der p 2–TC–Fc stained with Coomassie Blue (Abcam, UK). (**D**) Western blot detection of Der p 2–TC–Fc using anti-human IgG Fc antibody.

The plant-produced Der p 2-FL-Fc and Der p 2-TC-Fc proteins were purified from total soluble protein extracts using protein A column purification. Then the purified proteins were analyzed by SDS–PAGE, which revealed distinct bands corresponding to the expected molecular weight of Der p 2-FL-Fc (45 kDa) and Der p 2-TC-Fc (37 kDa) under reducing conditions (Figure 3A,C; original uncropped gels are provided in Supplementary Figure S2). The Western blot analysis using goat anti-human IgG Fc-HRP confirmed the identity of both fusion proteins (Figure 3B,D; original uncropped gels are provided in Supplementary Figure S2). The results demonstrated the expression and efficient purification of both Der p 2 variants from *N. benthamiana* leaves. The purified proteins showed high purity and integrity, which were further used for immunological evaluation.

### 3.3. Animal Experiments

**Figure 4:**
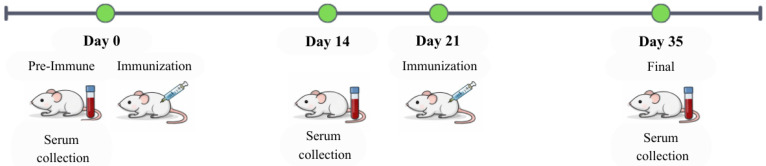
Immunization schedule for evaluating Der p 2-specific antibody response in BALB/c mice. Mice were immunized intramuscularly on days 0 and 21 with 10 µg of purified Der p 2–FL–Fc or Der p 2–TC–Fc formulated with alum adjuvant. Serum samples were collected on days 0, 14, and 35.

**Figure 5:**
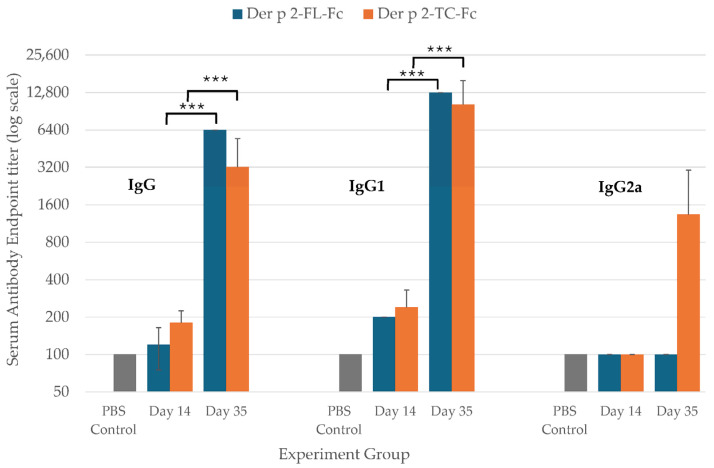
Antigen-specific serum IgG, IgG1, and IgG2a endpoint titers were determined by ELISA in mice immunized with Der p 2 full-length Fc fusion (Der p 2-FL-Fc) or truncated Der p 2 Fc fusion (Der p 2-TC-Fc). Serum samples were collected on day 14 and day 35 post-immunization. PBS-immunized mice served as negative controls. Antibody endpoint titers were presented on a log10 scale as mean ± SD (*n* = 5 per group). Statistical analysis was performed using two-way repeated-measures ANOVA, with time as the within-subject factor and construct as the between-subject factor. *** *p* < 0.001 denotes a significant increase compared with day 14 within the same group. No significant differences (*p* > 0.05) were observed between constructs at any time point.

The immunogenicity of the plant-produced Der p 2-Fc fusion proteins were assessed by immunizing the mice with purified Der p 2-FL-Fc or Der p 2-TC-Fc formulated with alum adjuvant (Figure 4). The serum samples collected at day 0 (pre-immune), 14 and 35 were analyzed for Der p 2-specific antibody responses by ELISA. The results showed that the mice immunized with both Der p 2-FL-Fc and Der p 2-TC-Fc developed significant Der p 2-specific IgG responses compared with PBS control group (Figure 5).

For total IgG, two-way repeated-measures ANOVA revealed a significant main effect of time (*F*_1,8_ = 60.46, *p* < 0.001, partial η^2^ = 0.883), indicating a significant increase in IgG titer from day 14 to day 35. There was no significant main effect of construct (*F*_1,8_ = 1.39, *p* = 0.273) nor a significant construct × time interaction (*F*_1,8_ = 4.11, *p* = 0.077). Bonferroni-adjusted pairwise comparison confirmed that the IgG titers were significantly higher at day 35 than at day 14 (*p* < 0.001). For IgG1, a significant main effect of time was observed (*F*_1,8_ = 58.15, *p* < 0.001, partial η^2^ = 0.879), with titers increasing significantly from day 14 to day 35. No significant main effect of construct (*F*_1,8_ = 0.67, *p* = 0.436) or construct x time interaction (*F*_1,8_ = 1.38, *p* = 0.274) was detected. In contrast, IgG2a responses showed no significant main effects of time (*F*_1,8_ = 2.67, *p* = 0.141), construct (*F*_1,8_ = 2.67, *p* = 0.141), or interaction (*F*_1,8_ = 2.67, *p* = 0.141).

These results demonstrated that the plant-produced Der p 2-Fc fusion proteins are immunogenic and induced strong antigen-specific humoral immune responses in mice, characterized by significant increase in total IgG and IgG1 over time.

## 4. Discussion

HDM allergy remains a major global health concern across all age groups, affecting 65–130 million people worldwide [41]. The major HDM species such as *Dermatophagoides pteronyssinus*, *Dermatophagoides farinae*, *Dermatophagoides microceras*, and *Blomia tropicalis* produce a wide range of allergenic proteins capable of triggering allergic responses and asthma. Among HDM allergens, Der p 2 is one of the most clinically relevant components due to its high prevalence of IgE sensitization [42]. The development of safer, standardized, and well-defined allergen preparations is a key priority in allergen immunotherapy. The recent advances in immunotherapy and molecular technology have emphasized the shift from crude allergen extracts toward purified recombinant allergens, which allow improved standardization, safety, and personalization of therapy [43,44].

The recombinant production of Der p 2 in different expression systems has been reported in earlier studies. Der p 2 produced in *E. coli* accumulates in inclusion bodies whereas the modified constructs with fusion tags enabled the soluble expression and resulted in secondary structure and IgE reactivity comparable to the natural allergen [45]. In another study, recombinant Der p 2 was produced in *E. coli* and *Pichia pastoris*, and both were compared. The yeast-secreted Der p 2 exhibited differences in structural features compared to the *E. coli* counterparts. However, both retained similar IgE recognition [46]. Proper folding and solubility can present challenges in certain recombinant systems depending on construct design and expression conditions; however, optimized bacterial and yeast platforms have produced properly folded Der p 2 in previous studies. In addition to established microbial platforms, plant-based expression systems have gained increasing attention as an alternative, particularly for applications requiring rapid production and eukaryotic processing.

The transient expression in *N. benthamiana* has emerged as an attractive platform for the rapid production of recombinant proteins, including vaccine antigens and therapeutic antibodies. The U.S. Food and Drug Administration (FDA) approval of the plant-derived enzyme Elelyso (taliglucerase alfa) and Elfabrio (pegunigalsidase alfa) developed by Protalix BioTherapeutics highlighted the potential of plant molecular farming [47,48,49]. In the recent decade, plant-based transient expression demonstrated its capability for rapid vaccine development. Companies such as Medicago Inc. (Canada), iBio (USA), Baiya Phytopharm Co., Ltd. (Thailand) and Kentucky BioProcessing Inc. (USA) developed COVID-19 vaccines within few months of the virus outbreak in 2020. Notably, the plant-derived vaccine developed by Medicago was tested in a phase 3 clinical trial across 85 centers worldwide and showed efficacy against COVID-19 variants [50]. These outcomes led to the regulatory approval of the vaccine by Health Canada on 24 February 2022 [51]. The approval of this vaccine represented a significant milestone for plant-based pharmaceuticals and increased public and industry recognition of the role of plants in next-generation vaccine development.

In the present study, we demonstrated the transient expression of *Dermatophagoides pteronyssinus* Der p 2 in *N. benthamiana* which provides a proof of concept for the production of recombinant HDM allergens in plants for potential immunotherapy applications. Compared with conventional systems that require fermentation optimization and scale-up development, transient plant expression enables rapid production, providing purified protein within a short timeframe. Although specific studies on *N. benthamiana*-derived Der p 2 remain limited, plant-derived allergens have been shown to modulate immune responses and reduce allergic inflammation in vivo [30,31,52]. In a recent study [53] the major birch pollen allergen Bet v 1 was expressed using a geminiviral vector–based transient expression system in plants. The recombinant protein accumulated up to 1.2 mg per gram of fresh leaf mass within five dpi. Importantly, the plant-produced Bet v 1 displayed immunological properties against human IgE identical to those of recombinant Bet v 1 produced in *B. brevis* [53]. This supports our observation that Der p 2 expressed in *N. benthamiana* maintains immunological features which highlight the potential of the plant system for producing allergen proteins. Our results showed that both Der p 2-FL-Fc and Der p 2-TC-Fc proteins accumulated to detectablelevels within a short time following agroinfiltration, with peak expression observed at 6 dpi, consistent with the earlier reports using geminiviral vector system [54]. The estimated accumulation levels (up to 62.4 µg/g fresh leaf weight) fall within the range commonly reported for this system. Although absolute yields obtained from optimized microbial fermentation can reach higher levels, the rapid transient production and simplified scalability of the plant platform provide practical advantages for early-stage development and rapid prototyping. The appearance of localized necrosis at infiltration sites is commonly reported in high-level transient expression studies and might be associated with endoplasmic reticulum stress [55,56]. Both full-length and truncated Der p 2 Fc-fusion proteins were successfully purified and exhibited the expected molecular weight and immunoreactivity as confirmed by SDS–PAGE and Western blot analysis. The incorporation of an Fc fusion tag was intended to enhance protein stability, facilitate purification and prolong serum half-life [57,58]. Collectively, these findings support the feasibility of producing recombinant Der p 2 in *N. benthamiana* using a transient expression system and provide a foundation for further optimization toward plant-based allergen production for therapeutic application.

The immunological evaluation in mice demonstrated that the plant-produced Der p 2-Fc fusion proteins elicited significant Der p 2-specific IgG responses following immunization. The results revealed a significant main effect of time, indicating that antibody titers increased following booster immunization. No significant main effect of construct or construct × time interaction was observed, suggesting that the full-length and truncated constructs elicited comparable humoral immune responses under the experimental conditions tested. IgG1 responses showed a similar time-dependent increase, whereas IgG2a responses did not differ significantly among groups. The significant increase in antibody titers after the booster immunization supports the immunogenic potential of plant-derived Der p 2. This highlights the feasibility of using molecularly defined plant-produced allergens for immunotherapy, an approach increasingly explored in next-generation allergy vaccines.

Although the present study establishes a solid proof of concept, further investigations are needed to fully assess the clinical relevance of plant-produced Der p 2. The present study assessed immunogenicity through antigen-specific IgG and IgG subclass responses; additional evaluation of Der p 2-specific IgE responses would be important to better determine the allergenic potential of the antigen. The future studies should focus on comprehensive in vitro and in vivo evaluation studies to assess safety and efficacy in established allergy models. Additionally, optimization of expression strategies to reduce plant tissue necrosis and improve protein yield will further enhance the applicability of this system.

## 5. Conclusions

This study demonstrates the successful transient expression of Der p 2-Fc fusion proteins in plant expression system and highlights its potential as a rapid platform for producing recombinant allergens. The results provide a solid foundation for further in vitro studies and subsequent preclinical evaluation. These findings contribute to the increasing evidence supporting that plant molecular farming is a promising alternative platform for recombinant protein production and provide a basis for further studies in allergy immunotherapy.

## Data Availability

Data supporting the findings of this study are available from the corresponding author upon request.

## Supplementary Materials

The following supporting information can be downloaded at: https://www.mdpi.com/article/10.3390/vaccines14030256/s1, Figure S1: Densitometric analysis of Der p 2-FL-Fc and Der p 2-TC-Fc, including normalized intensity data, regions of interest (ROIs) used for quantification, densitometry graphs, and uncropped Western blot images for day optimization studies; Figure S2: Uncropped original SDS–PAGE gels and Western blot films.
