# Supplementary material for: Transient Expression and Immunogenicity Assessment of the Dermatophagoides pteronyssinus Der p 2 Allergen Produced in Nicotiana benthamiana
Source: Vaccines (Basel). 2026 Mar 11;14(3):256. doi: 10.3390/vaccines14030256 (PMC13030849; doi:10.3390/vaccines14030256)

Supplementary data

Supplementary Figure S1:

Densitometric analysis of Der p2-FL-Fc and Der p2-TC-Fc Western blots, expressed as percentage of D2.

Lane	Sample	Day post-infiltration	Raw volume (Integrated Density)	Normalized Intensity (% of D2)
Der p2-FL-Fc				
L1	D2	2	39357.77	100%
L2	D4	4	16255418	413%
L3	D6	6	28355888	720%
L4	D8	8	16130774	410%
Der p2-TC-Fc				
L1	D2	2	219223.67	100%
L2	D4	4	448946.18	205%
L3	D6	6	633386.17	289%
L4	D8	8	447011.57	204%

Area measurement of Der p2-FL-Fc and Der p2-TC-Fc, respectively, showing the regions of interest (ROIs) used for densitometric quantification.

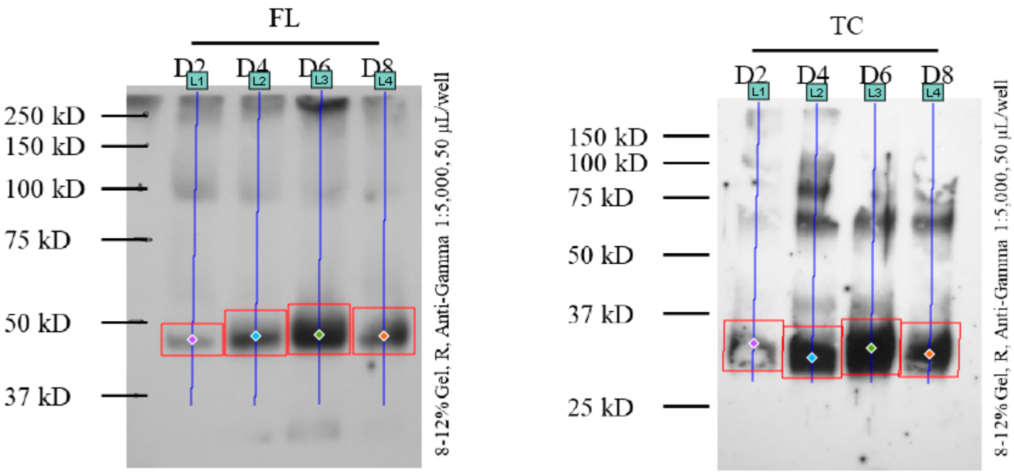

Densitometry graph of Der p2-FL-Fc in each day post-infiltration, generated using ImageQuant TL software to calculate integrated density.

- Der p2-FL-Fc (D2)

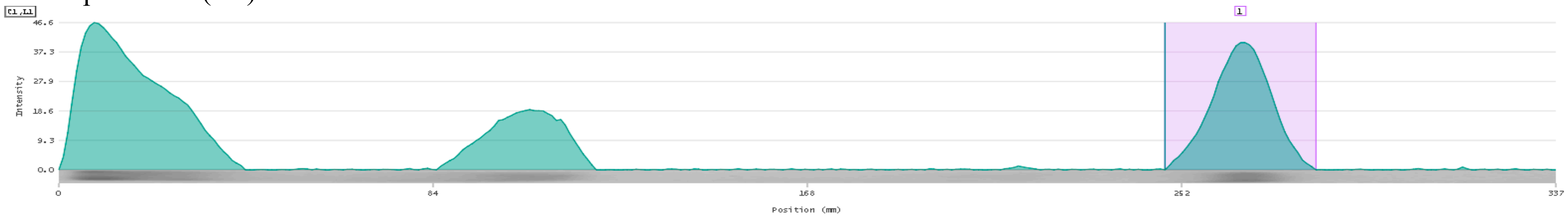

- Der p2-FL-Fc (D4)

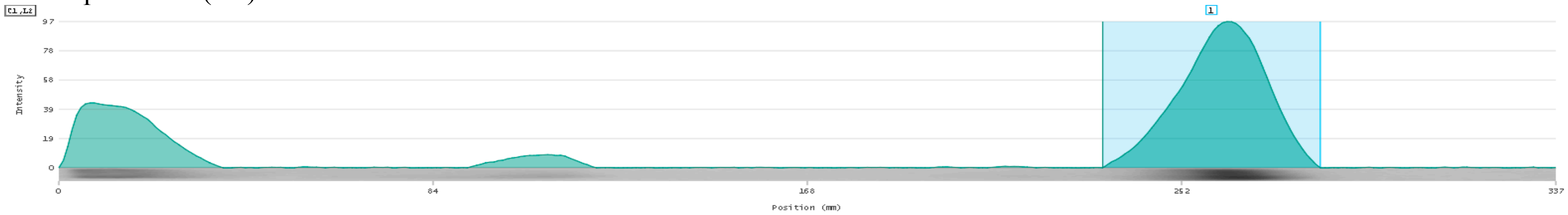

- Der p2-FL-Fc (D6)

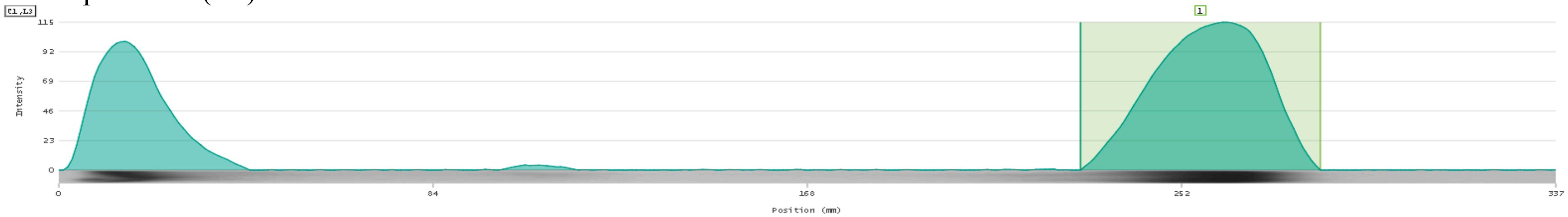

- Der p2-FL-Fc (D8)

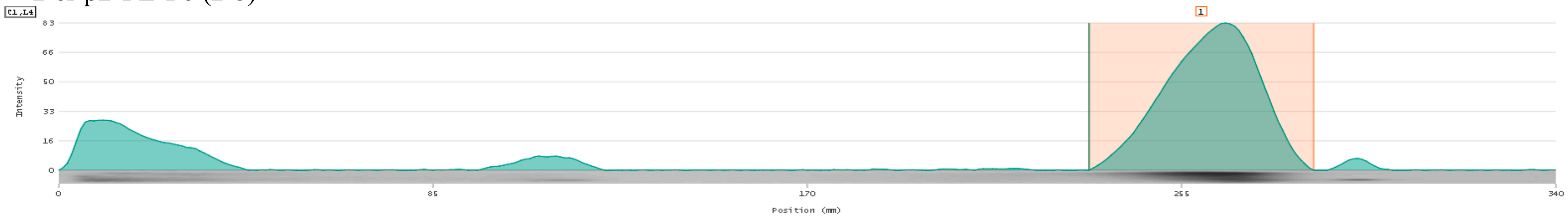

Densitometry graph of Der p2-TC-Fc, generated using ImageQuant TL software to calculate integrated density.

- Der p2-TC-Fc (D2)

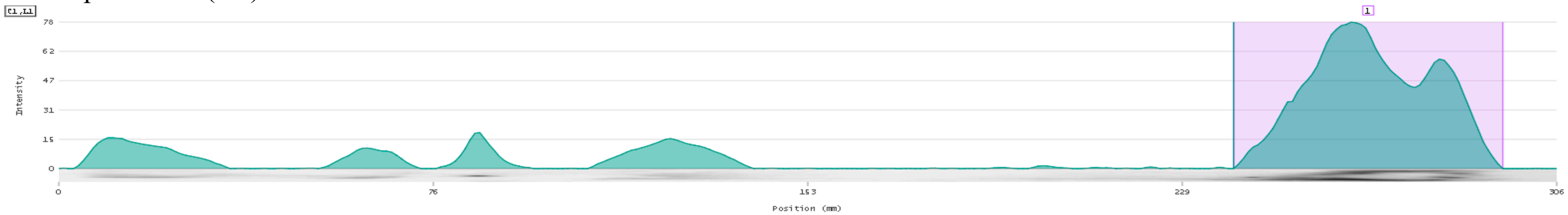

- Der p2-TC-Fc (D4)

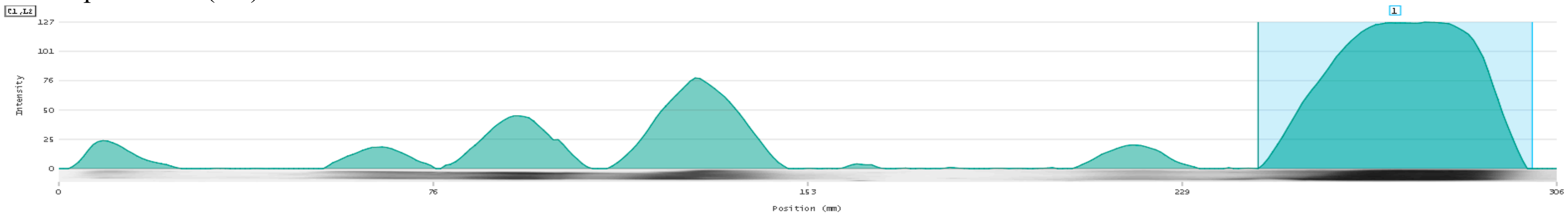

- Der p2-TC-Fc (D6)

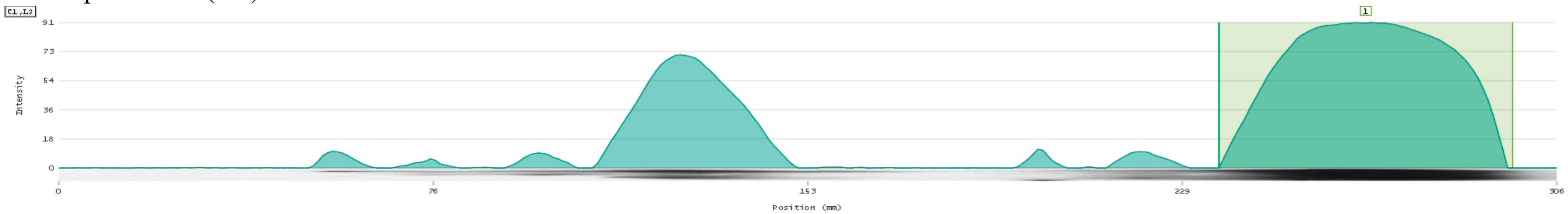

- Der p2-TC-Fc (D8)

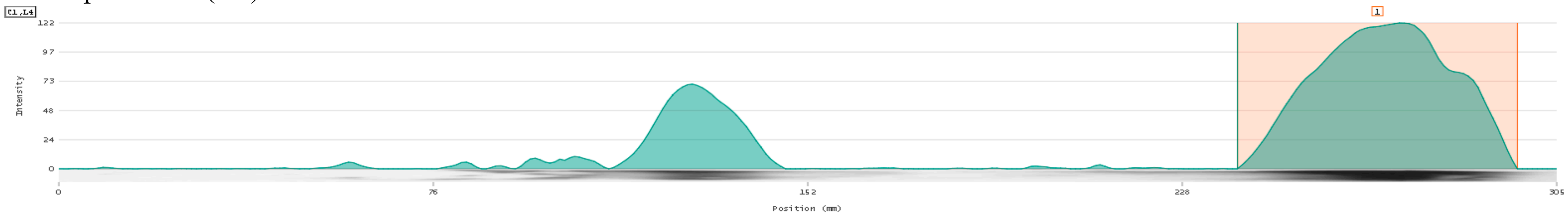

Western blot film showing the membranes used for day optimization of Der p2-FL-Fc and Der p2-TC-Fc, respectively

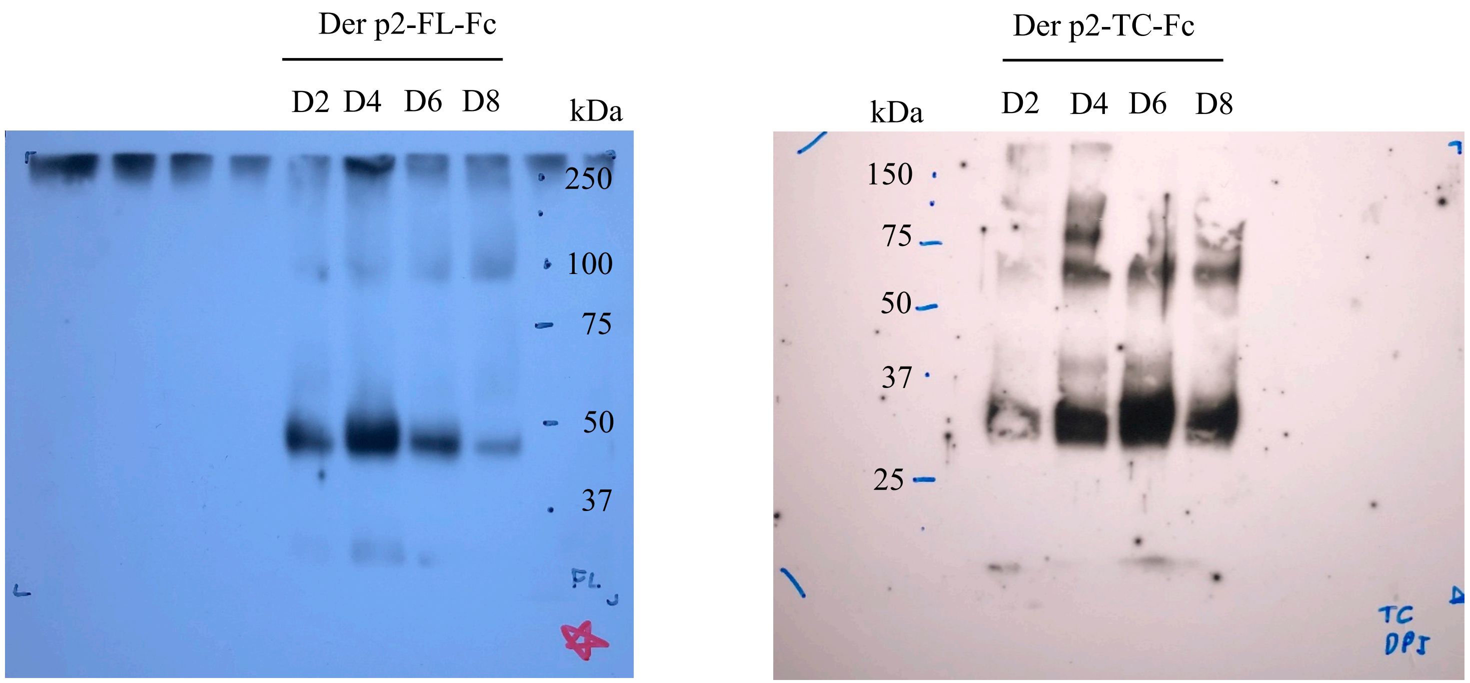

Supplementary Figure S2:

SDS-PAGE gel of Figure 3A for Der p2-FL-Fc

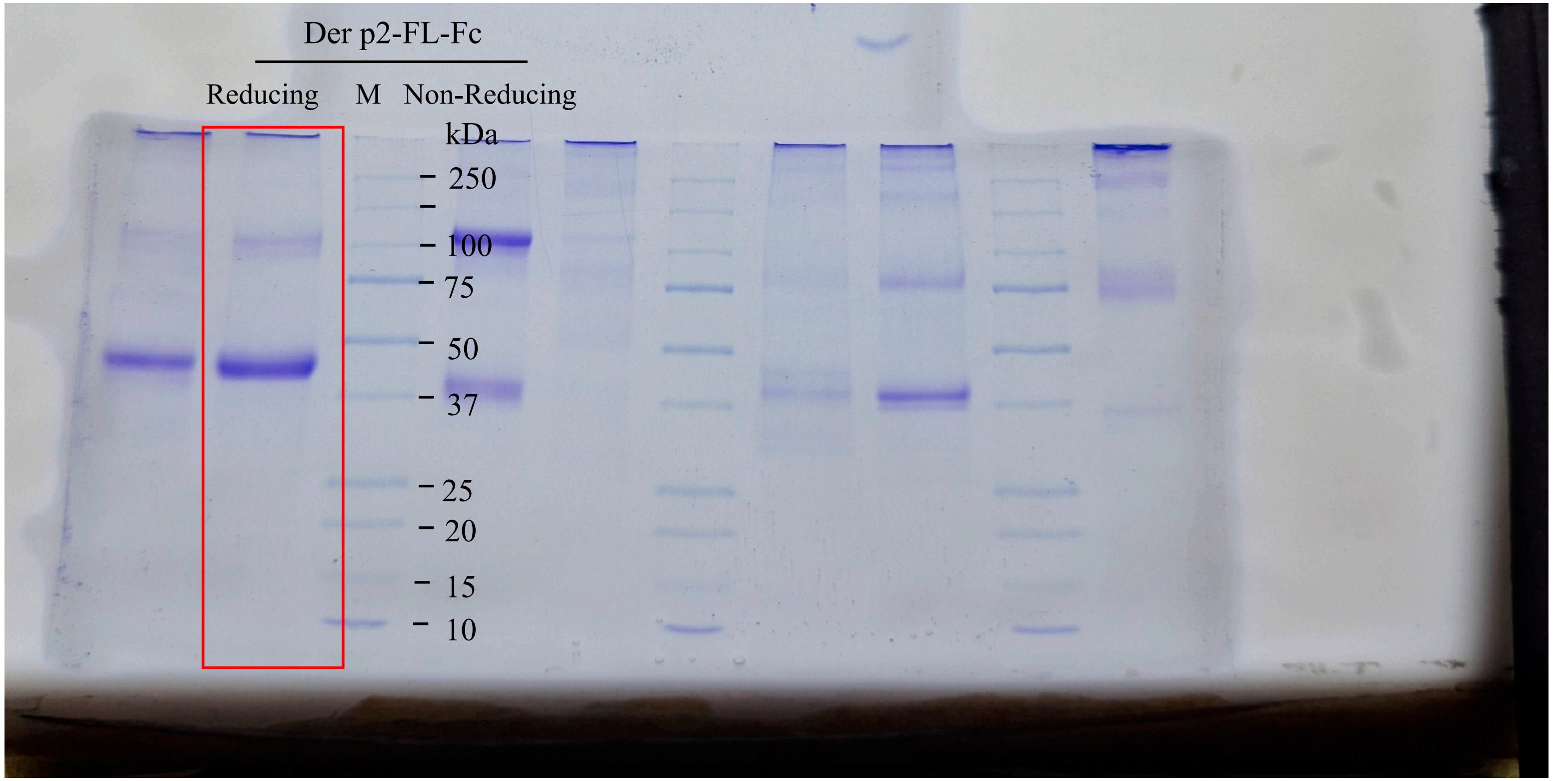

Western blot film of Figure 3B for Der p2-FL-Fc

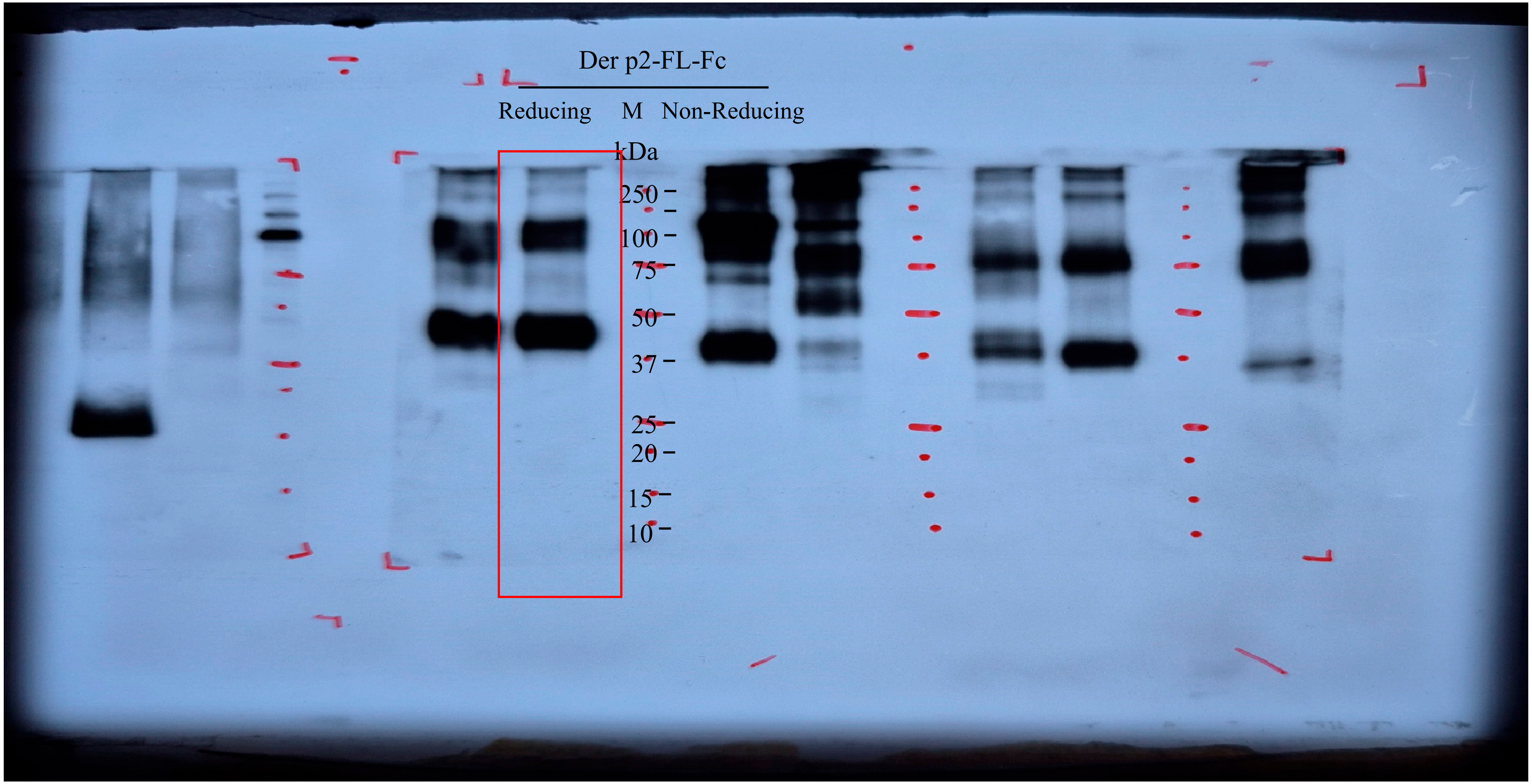

SDS-PAGE gel of Figure 3C for Der p2-TC-Fc

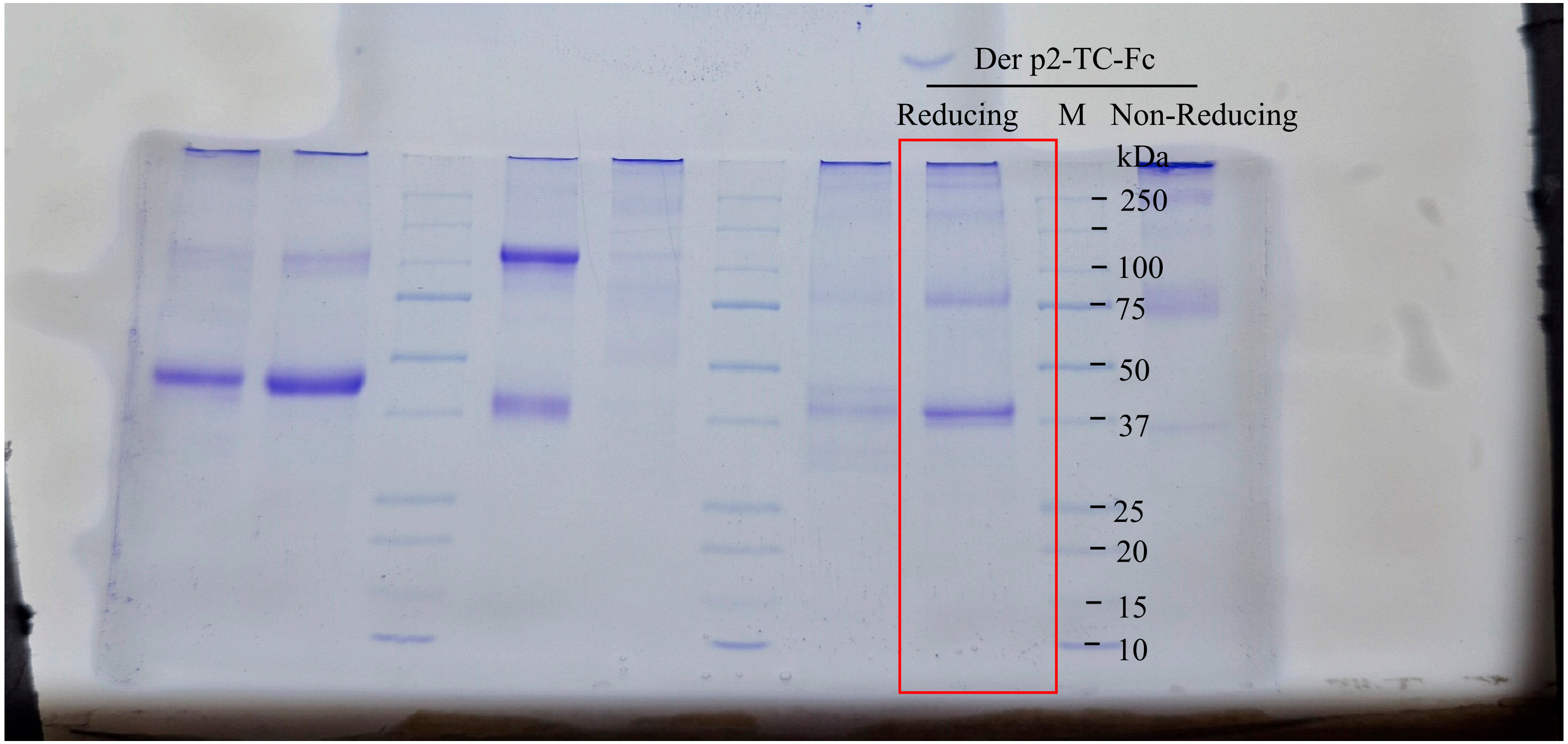

Western blot film of Figure 3D for Der p2-TC-Fc

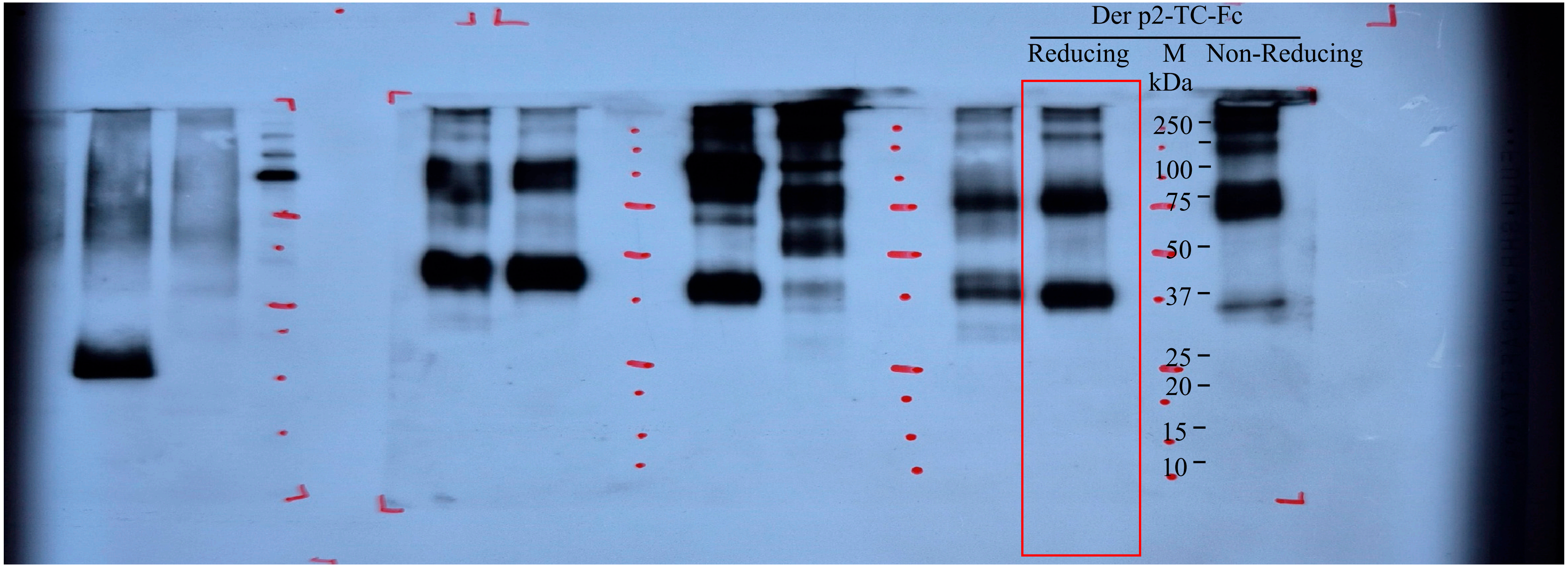

Supplement: Supplementary file 1 [file vaccines-14-00256-s001.zip › vaccines-4156836-supplementary.pdf]
